# Discovery of a selective dual-specificity tyrosine phosphorylation-regulated kinase 1B inhibitor with anti-adipogenic and anti-diabetic activities

**DOI:** 10.3389/fphar.2025.1645033

**Published:** 2025-07-29

**Authors:** Sein Kang, Yoon-Ju Na, Kyoung Jin Choi, Won Hoon Jung, Areum Park, Jeonghui Im, Sung Bum Park, Byumseok Koh, Joo-Youn Lee, Kwang-Lae Hoe, Heung Jae Kim, Sang Joon Shin, Hyuk Lee, Ki Young Kim

**Affiliations:** ^1^Therapeutics and Biotechnology Division, Korea Research Institute of Chemical Technology, Daejeon, Republic of Korea; ^2^Graduate School of New Drug Discovery and Development, Chungnam National University, Daejeon, Republic of Korea; ^3^ Therasid Bioscience Co Ltd., Seongnam-si, Gyeonggi-do, Republic of Korea; ^4^Department of Medicine, Yonsei University College of Medicine, Seoul, Republic of Korea

**Keywords:** DYRK1B, adipogenesis, obesity, type 2 diabetes, FOXO1A

## Abstract

**Background:**

Dual-specificity tyrosine phosphorylation-regulated kinase 1B (DYRK1B) is implicated in metabolic diseases, with high expression linked to adipocyte differentiation and metabolic disorders. This study investigated the anti-adipogenic and anti-diabetic effects of a novel selective DYRK1B inhibitor, N-(4-(3-(4-methoxyphenyl)-1H-pyrazolo [3,4-b]pyridin-5-yl) phenyl)acetamide (KS-40070).

**Methods:**

The efficacy of KS-40070 was evaluated using 3T3-L1 cells, adipose-derived mesenchymal stem cells (ADMSC), and diet-induced obesity (DIO) mice.

**Results:**

Treatment with KS-40070 dose-dependently inhibited 3T3-L1 preadipocyte differentiation, reducing key adipogenic transcription factors like PPARγ and C/EBPα, along with related proteins. KS-40070 suppressed lipid accumulation by decreasing Akt-FOXO1A signaling and GSK3β expression. Importantly, these effects were abolished in DYRK1B knockdown cells, confirming DYRK1B's role. In DIO mice, KS-40070 suppressed body weight gain, food consumption, serum lipid levels, and adipose tissue mass. It also improved insulin resistance and glucose intolerance.

**Conclusion:**

These findings suggest that inhibiting DYRK1B with agents like KS-40070 presents a promising therapeutic strategy for obesity and type 2 diabetes.

## Highlights

• DYRK1B inhibitor KS-40070 suppresses adipocyte differentiation in 3T3-L1 cells and ADMSCs.
• KS-40070 reduces lipid accumulation via Akt-FOXO1A and GSK3β pathway modulation.
• DYRK1B knockdown abolishes KS-40070 effects, confirming its role in adipogenesis.
• KS-40070 improves insulin sensitivity and glucose tolerance in DIO mice.
• DYRK1B inhibition presents a potential therapeutic strategy for obesity and diabetes.

## 1 Intruduction

Metabolic syndrome, encompassing conditions such as obesity, type 2 diabetes mellitus, and fatty liver disease, is characterized by dysregulation in multiple cellular signaling pathways and molecular mechanisms. One such pathway gaining attention in metabolic regulation is governed by the dual-specificity tyrosine phosphorylation-regulated kinase 1B (DYRK1B).

DYRK1B, a member of the DYRK family of protein kinases, participates in diverse cellular processes including cell proliferation, differentiation, and survival (Widowati et al., 2018). It has been implicated in regulating adipogenesis, glucose metabolism, and insulin sensitivity, rendering it a potential target for therapeutic intervention in metabolic diseases. DYRK1B exhibits high expression in tissues relevant to metabolic regulation, such as adipose tissue, liver, and skeletal muscle. Notably, in adipocytes, DYRK1B is involved in the differentiation of preadipocytes into mature adipocytes, a critical process for maintaining adipose tissue function and glucose homeostasis (Kokkorakis and Gaitanou, 2020).

Metabolic syndrome arises from complex regulatory dysfunctions, with the PI3K/AKT/mTOR signaling network serving as a central axis that regulates adipogenesis, glucose metabolism, and insulin signaling. Recent studies emphasize its pivotal role in the development of obesity-related metabolic disorders and its potential as a therapeutic target (Chu H. et al., 2022; Omolekan et al., 2024). Key downstream factors like FOXO1A and GSK3β contribute to lipid accumulation and insulin resistance (Teaney and Cyr, 2023). Recent studies also highlight the role of bile acid signaling via FXR and TGR5 in improving metabolic balance (Chiang and Ferrell, 2020; Huang et al., 2024). Recent studies have revealed that mutations in DYRK1B linked to metabolic syndrome impair the chaperone-mediated maturation of its kinase domain, thereby disrupting its regulatory role in metabolic pathways (Abu Jhaisha et al., 2017). In this context, advances in heteroaryl medicinal chemistry have facilitated the development of selective kinase inhibitors capable of precisely targeting emerging regulators such as DYRK1B, underscoring its therapeutic potential in addressing the multifactorial nature of metabolic disorders.

Research has also identified genetic variations and mutations in the DYRK1B gene associated with metabolic disorders. For example, gain-of-function mutations in DYRK1B have been linked to familial forms of obesity and metabolic syndrome, highlighting the importance of DYRK1B in regulating metabolic pathways (Keramati et al., 2014; Abu Jhaisha et al., 2017). Additionally, DYRK1B plays a role in hepatic glucose production regulation. Dysregulation of DYRK1B activity in the liver has been shown to affect glucose metabolism, leading to increased gluconeogenesis and elevated blood glucose levels, characteristic features of type 2 diabetes mellitus (Bhat et al., 2022; Bhat and Mani, 2023). Overall, research into the role of DYRK1B in metabolic diseases is ongoing and continues to uncover its significance in the pathophysiology of obesity, diabetes, and related metabolic disorders. Targeting DYRK1B and its associated pathways may hold promise for the development of novel therapeutic strategies for treating metabolic diseases and improving metabolic health.

In our previous study, a series of 1H-pyrazolo [3,4-b]pyridine derivatives were synthesized and evaluated for their ability to inhibit DYRK1B. Among these derivatives, (N-(4-(3-(4-methoxyphenyl)-1H-pyrazolo [3,4-b]pyridin-5-yl)phenyl)acetamide (KS-40070) exhibited most potent inhibitory effects (Park et al., 2021).

In this study, we investigated to identify potential therapeutic effects and underlying mechanisms of a novel selective DYRK1B inhibitor, KS-40070, using 3T3-L1 adipocytes, human ADMSC, and DIO mice model.

## 2 Materials and methods

### 2.1 Synthesis of a novel DYRK1B inhibitor, KS-40070

KS-40070 is synthesized in the Korea Research Institute of Chemical Technology (Park et al., 2021). The purity of KS-40070 was determined to be 99.9%. Analytical evaluation was conducted using a Waters ACQUITY H-Class PLUS UPLC system (Waters Corporation, Milford, MA, United States) coupled with an SQD2 mass spectrometry detector. Chromatographic separation was performed on an ACQUITY UPLC BEH C18 column (1.7 μm, 2.1 × 50 mm). The mobile phase consisted of buffer A (water containing 0.1% formic acid) and buffer B (chromatographic-grade acetonitrile containing 0.1% formic acid).

### 2.2 Cell culture and differentiation

3T3-L1 cells (ATCC #CL-173, mouse preadipocyte) were purchased from the American Type Culture Collection (Manassas, VA, United States). 3T3-L1 cells were seeded 5 × 10^5^ in 6-well plates coated with collagen (Corning, Corning, NY, United States) and maintained in Dulbecco’s modified Eagle’s medium (DMEM, Gibco/Invitrogen, Carlsbad, CA, United States) supplemented with 10% fetal bovine serum (FBS), 100 μg/mL penicillin, and 100 μg/mL streptomycin. Cells were grown at 37°C in a 5% CO_2_ incubator with 95% humidified air. After cells reached complete confluence, cells were treated with differentiation-inducing cocktail, DMI medium containing dexamethasone (DEX, final concentration 2.5 μΜ), isobutyl-1-methylxanthine (IBMX, final concentration 0.5 mM), insulin (final concentration 2 μg/mL) and 3–4 days that was then replaced with insulin (2 μg/mL) for another 4 days. Medium was replaced every 2 days.

Human adipose-derived mesenchymal stem cells (ADMSC; CB-ADMSC, # CEFO-ADMSC) were used to generate 3D human adipose tissue-mimicking model, as previously described by Park et al. (Park et al., 2020). Brefly, ADMSCs were purchased from CEFO Bio (Seoul, Korea) and maintained using a Human Adipose-derived MSC Kit (CEFO Bio) supplemented with 100 μg/mL penicillin and 100 μg/mL streptomycin. Cells were grown at 37°C in a 5% CO_2_ incubator with 95% humidified air. After cells reached complete confluence, they were treated with DMIT cocktail containing DEX (final concentration 10 μΜ), IBMX (final concentration 1 mM), insulin (final concentration 10 μg/mL), and indomethacin (final concentration 40 μΜ) for 12 days. Medium was replaced every 2 days. Alginate beads for 3D cell culture were produced by mixing ADMSC, alginate (2%, w/v), gelatin (0.5%), collagen (40 μL/mL), and serum free medium that was subsequently incubated for 30 min at 37°C. Cells suspended in alginate solution were transferred by syringe with an 18G needle into 50 mL tubes containing 1% calcium chloride. Excess calcium chloride was removed by incubating alginate beads in serum free medium for about 5 min. Medium was replaced and transferred to a 25 T flask and then incubated overnight for stabilization. The next day, alginate beads were transferred to 6-well plates, and differentiation was initiated. All cell lines were used passage under 6 (At higher 6 passage, the lipid differentiation rate is decreased.)

### 2.3 Western blot analysis

Cells were washed twice with Dulbecco’s Phosphate Buffered Saline (DPBS) and RIPA lysis buffer (Thermo Fisher Scientific, Waltham, MA, United States) containing a protease inhibitor cocktail tablet (Sigma Aldrich, St. Louis, MO, United States) and phosphatase inhibitor cocktail tablet (Roche, Basel, Switzerland) were added for lysis. Whole cell lysates were centrifuged at 13,000 rpm, after which supernatants were collected. Protein concentration in samples was determined using a BCA protein assay kit (Thermo Fisher Scientific). Then, 20 µg of total protein was loaded into a Bolt 4%–12% Bis-Tris Gel (Invitrogen) and transferred to a PVDF membrane (Merck Millipore, Burlington, MA, United States). Blocked membranes were incubated with antibodies. Protein bands were visualized with the Super Signal West Femto Maximum Sensitivity Substrate kit (Thermo Fisher Scientific) and analyzed by chemiluminescence (Biorad, Hercules, CA, United States). DYRK1B, DYRK1A, PPARγ, C/EBPα, perilipin, FABP4, adiponectin, phospho-Akt, Akt, phospho-FOXO1A, phospho-GSK α/β, GSK3α/β and β-actin were purchased from Cell Signaling Technology (Danvers, MA, United States). FOXO1A, Glucose-6-phosphatase (G6Pase) and tumor necrosis factor-α (TNF-α) were purchased from Abcam (Cambridge, UK). Western blot density results were analyzed with ImageJ software provided by the National Institutes of Health (Bethesda, MD, United States).

### 2.4 Knockdown study

DYRK1B siRNA and negative control siRNA were purchased from Thermo Fisher Scientific. 3T3-L1 cells were transfected using the Lipofectamine RNAiMAX reagent (Thermo Fisher Scientific) according to the manufacturer’s protocol. Final concentration of siRNA was 250 pmol. After transfection, cells were treated with DMI containing insulin (final concentration 2 μg/mL), IBMX (final concentration 0.5 mM), and DEX (final concentration 2.5 μΜ) for 2 days that was then replaced with insulin (2 μg/mL) for another 4 days. Medium was replaced every 2 days.

### 2.5 DYRK1B inhibitory activity assay

A LanthaScreen Eu Kinase Binding Assay (Invitrogen) was performed according to the manufacturer’s protocol. Briefly, compounds were diluted into kinase buffer A. Tracer solution in kinase and kinase/antibody solutions were then prepared, mixed with the compound, and incubated for 1 h at room temperature. Acceptor/tracer emission (665 nm) and antibody/donor emission (615 nm) were measured and then used to calculate the emission ratio. Human DYRK1B recombinant protein (#PV4649), human DYRK1A recombinant protein (#PV3785), human DYRK2 recombinant protein (#PV6331), 5× kinase buffer A (#PV3189), kinase tracer 236 (#PV5592), and LanthaScreen™ Eu-anti-GST antibody (#PV5594) were purchased from Invitrogen.

### 2.6 Lipid droplet fluorescence staining

Lipid droplets were stained using boron-dipyrromethene (BODIPY) 493/503 (Invitrogen). Nuclei were stained using 4′,6-diamidino-2-phenylindole (DAPI, Cell Signaling Technology). For fixative, cells were washed with DPBS and then incubated in a formalin solution for 10 min. After removing the formalin solution with DPBS, cells were permeabilized using 0.25% Triton X-100 for 10 min. After permeabilization, solution was removed with DPBS. The cells were stained with BODIPY solution for 10 min. Stained cellular lipid droplets were washed with DPBS, and cells were then stained with DAPI solution for 1 min. Afterwards, cells were washed with DPBS and visualized using a fluorescence microscope.

Furthermore, lipid accumulation was estimated in living cells using an AdipoRed™ Adipogenesis Assay Reagent (PT-7009, Lonza, Basal, Switzerland). Cells were washed, AdipoRed solution (30 μL/mL in DPBS) was added, and cells were incubated for 20 min at room temperature. The 3 dimensional (3D) cells were transferred to micro tubes, and cells in the alginate beads were isolated using 16 mM EDTA. After shaking for 5 min, lysates were centrifuged at 13,000 rpm for 1 min 30 s. After removing the supernatant, the red pellet was suspended with DPBS, then it was transferred to a plate and measured using a microplate reader (Molecular Devices, San Jose, CA, United States) at an excitation wavelength of 485 nm and an emission wavelength of 538 nm. DNA was quantified using a Blue Fluorometric dsDNA Quantitation Kit (Invitrogen). Hoechst 33,342 with TNE buffer (1.25 μL/mL) was added to the plate, incubated for 5 min at room temperature, and measured using a microplate reader at an excitation wavelength of 355 nm and an emission wavelength of 460 nm.

### 2.7 Animal and administration

Four-week-old male (13–15 g) C57BL/6J mice were purchased from Orient Bio (Seongnam, Korea). All animal experiments were conducted according to the guidelines for animal experimentation and approved by the institutional animal care and use committee of KRICT (Approval number: 2019-7A-01–02). All animals were housed in plastic cages and allowed free access to water and food. They were maintained in a room illuminated daily from 07:00 to 19:00 (12/12 light/dark cycle) under controlled temperature (23°C ± 1°C), ventilation (10–12 air changes per hour), and humidity (55% ± 5%). At the end of the experiments, mice were euthanized using carbon dioxide inhalation in accordance with institutional guidelines for animal care and use.

Obesity/diabetes was induced in C57BL/6J mice by feeding a high fat diet (Diet Research Inc., New Brunswick, NJ, United States) with 60% of its energy from fat for 5 weeks starting at 5 weeks old. Rosiglitazone (Combi-Blocks, San Diego, CA, United States) and Xenical (Sigma-Aldrich) were used as reference compounds. Mice were divided into six groups (n = 7) and fed normal diet (ND, Lean), DIO (Diet-induced obesity mouse), DIO with 10 mg/kg rosiglitazone, DIO with 20 mg/kg Xenical, DIO with 50 mg/kg of KS-40070, and DIO with 100 mg/kg of KS-40070 for 9 days. All compounds were dissolved in 0.5% carboxymethyl cellulose and orally administered twice a day (10:00 and 17:00).

### 2.8 Analysis of plasma biochemical parameters concentration

Blood samples of mice (50 μL) were collected after overnight fasting from the retro-orbital plexus and stored in heparin-coated capillary tubes. Blood samples were immediately centrifuged for 15 min at 13,000 rpm and then stored at −20°C prior to assay. Concentrations of total cholesterol, high-density lipoprotein cholesterol (HDL-C), low-density lipoprotein cholesterol (LDL-C), triglyceride (TG), ALT, and AST were measured using a Response 920 automated biochemical analyzer (DiaSys, Holzheim, Germany).

### 2.9 Histological analysis

After mice were sacrificed, white adipose tissue (related tissues of metabolic disease, subcutaneous and visceral fat) was collected, fixed in 10% formalin, embedded in paraffin, and sectioned to a thickness of 5 μm. For hematoxylin and eosin (H&E) staining, sections were deparaffinized with xylene three times, rehydrated by pass -through in stepped concentrations of alcohol baths (100%, 90%, 80%, and 70%), and then washed briefly under running water. Sections were stained in hematoxylin solution (Sigma-Aldrich) for 15 min and washed under running water for 3 min, then incubated in bluing solution for 5 s. Sections were subsequently stained with eosin (Daejung Chemical, Siheung, Korea) for 3 s, washed under running water for 3 min, and then dehydrated via pass-through in stepped concentrations of alcohol baths (70%, 80%, 95%, and 100%), followed by clearing with xylene three times. Stained sections were mounted with mounting media (Fisher Chemical, Pittsburgh, PA, United States) and visualized using light microscopy (DS-Ri2, Nikon, Tokyo, Japan).

### 2.10 Oral Glucose Tolerance Test (OGTT), Insulin Tolerance Test (ITT), and HOMA-IR index analysis

For the OGTT, mice were orally administered 0.2 g/mL glucose (Sigma-Aldrich) after overnight fasting. Blood samples (50 μL) were obtained at 15, 30, and 60 min afterwards from the retro-orbital plexus and transferred to heparin-coated capillary tubes. For the ITT, non-fasted blood samples were drawn prior to insulin injection (0.55 U/kg, i.p.; Sigma-Aldrich, USA) and at 15, 30, and 60 min after insulin administration. The blood samples were immediately centrifuged for 15 min at 13,000 rpm and then stored at −20°C prior to assay. Glucose in plasma was measured using a Response 920 automated biochemical analyzer, (DiaSys, Holzheim, Germany). Plasma insulin levels were measured using an ultrasensitive mouse/rat insulin ELISA kit (FUJIFILM Wako Shibayagi corporation, Shibukawa, Japan). Homeostatic Model Assessment for Insulin Resistance (HOMA-IR) values to indicate insulin sensitivity were calculated based on the following formula: (fasting insulin [μU/mL]xfasting glucose [mg/dL])/405) (Ko et al., 2014).

### 2.11 Homology modeling

To build a three-dimensional (3D) structure of human DYRK1B was generated using SWISS-MODEL server (https://swissmodel.expasy.org/). The sequence of hDYRK1B was obtained from the UniProtKB database (https://www.uniprot.org; accession number Q9Y463). For a 3D template of hDYRK1B, sequence similarity search was performed using BLAST (Basic Local Alignment Search Tool) (Camacho et al., 2009) available on the website. The template X-ray crystal structure, hDYRK1A (PDB code 6EIF) was selected as the top score sequence with high resolution. The sequence identity and similarity between hDYRK1B and hDYRK1A are 83.3% and 92.8% respectively.

### 2.12 Molecular docking

To predict the binding mode of compound, KS-40070, we performed a docking study using Schrodinger Suite 2020–4 (Schrödinger, LLC, New York, NY, United States, 2020). The DYRK1B 3D structure was obtained from homology modeling, and the protein preparation was revised using Protein Preparation Wizard in Maestro. The receptor grid box for docking was generated 25 Å × 25 Å × 25 Å size centered on complexed ligand, which was copied from the superimposition of hDYRK1A in complex with inhibitor. Compounds were minimized using an OPLS3e force field with a dielectric constant of 80.0 in MacroModel v13.0. Molecular docking of compounds was performed using the Glide v8.9 with SP method. The proposed binding models of compounds for DYRK1B were represented using Discovery Studio 2020 (Dassault Systèmes BIOVIA, San Diego, CA, United States, 2020).

### 2.13 Statistics

Graphical results are presented as mean ± standard error of the mean (S.E.M). Statistical significance was analyzed using GraphPad Prism software (GraphPad Software Inc., La Jolla, CA, United States) and determined by student’s *t-*test or a one-way analysis of variance followed by Tukey’s multiple-comparison test. A *P-*value of less than 0.05 was considered statistically significant.

## 3 Results

### 3.1 Increased DYRK1B protein expression in differentiated 3T3-L1 cells

**FIGURE 1:**
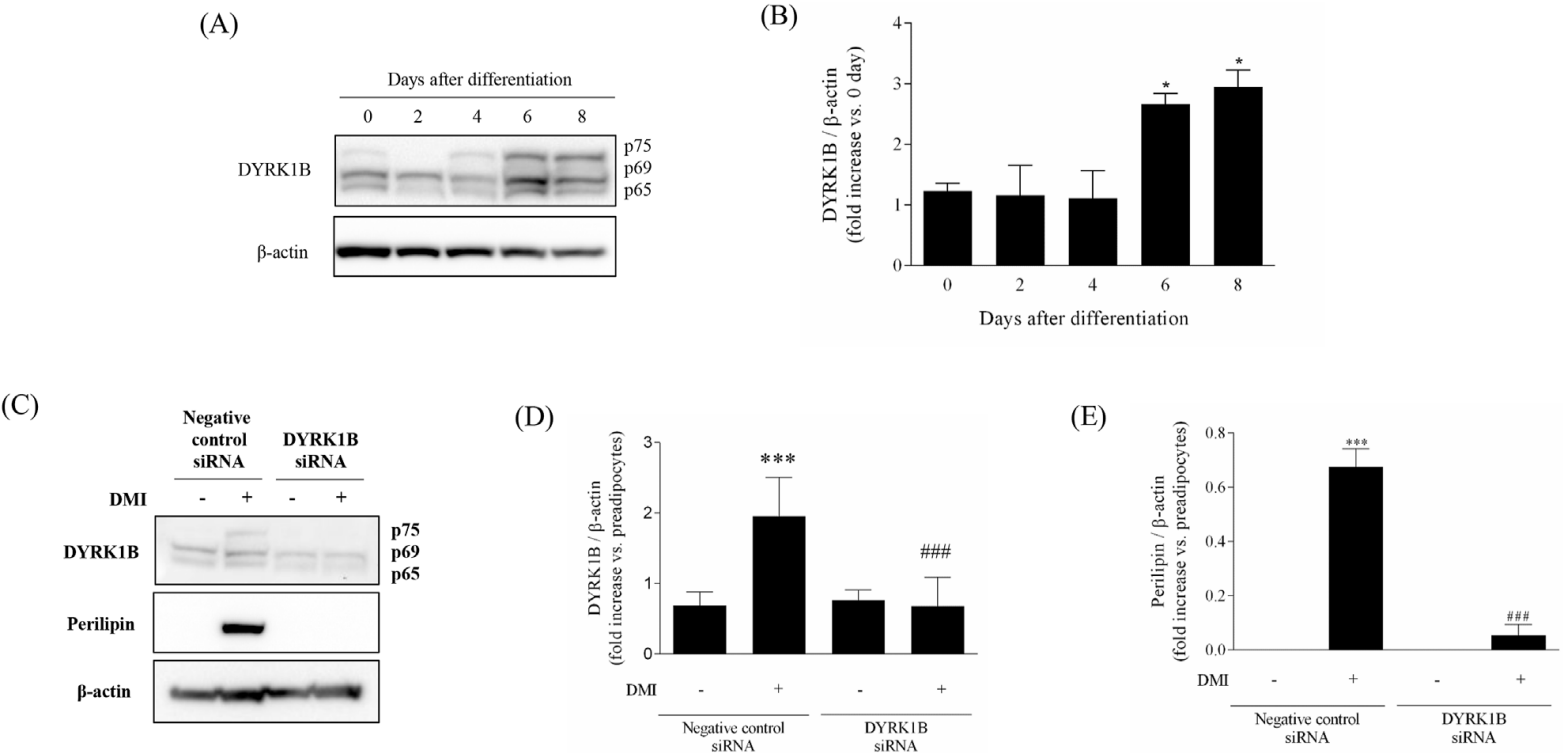
Effect of DYRK1B on adipogenesis in 3T3-L1 cells. **(A)** DYRK1B expression levels as assayed by Western blot. **(B)** Quantification of band intensities with ImageJ software. **(C)** DYRK1B and lipid-droplet related protein expression levels by siRNA-mediated silencing of dyrk1b in 3T3-L1 cells. **(D)** The amount of DYRK1B was calculated relative to β-actin protein levels. **(E)** The amount of Perilipin was calculated relative to β-actin protein levels. Quantification of band intensities with ImageJ software. Results are presented as mean ± S.E.M. (n = 3) of at least three independent experiments. ^*^ *P* < 0.05, ^***^ *P* < 0.001 vs. DMI (−) (preadipocytes); ^*###*^ *P* < 0.001 vs. DMI (+) (adipocytes).

We assessed the upregulation of DYRK1B proteins during adipogenesis by monitoring expression change throughout the differentiation process in 3T3-L1 cells via Western blot analysis. As depicted in Figures 1A,B, DYRK1B expression exhibited a progressive increase during adipogenesis, reaching saturation on 8 days of differentiation. Subsequently, to elucidate the effect of DYRK1B on adipocyte differentiation, we conducted a gene silencing assay. This assay revealed that the knockdown of DYRK1B not only led to a reduction in DYRK1B expression but also resulted in decreased levels of Perilipin, a protein associated with lipid droplet formation (Figures 1C–E) (Itabe et al., 2017). BODIPY staining further demonstrated a depletion in lipid droplet (Figures 4D,E). These results suggest that DYRK1B affects lipid formation and is related to obesity.

### 3.2 A novel selective DYRK1B inhibitor, KS-40070

**FIGURE 2:**
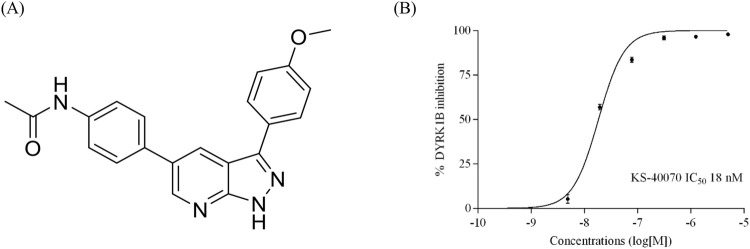
Structure and DYRK1B inhibitory activity of KS-40070. **(A)** Structure of KS-40070 **(B)** IC_50_ was measured using a LanthaScreen Kinase Binding Assay for DYRK1B and analyzed individually in triplicate. Results are presented as mean ± S.E.M. (n = 3).

We investigated a new class of DYRK1B inhibitors and developed KS-40070 (Figure 2A) (Park et al., 2021). KS-40070 exhibited strong inhibitory efficacy against DYRK1B, inhibiting the activity of DYRK1B by more than 95% at 5 μM, with an IC_50_ value of 18 nM (Figure 2B). To verify the selectivity of KS-40070 for DYRK, LanthaScreen Kinase Binding Assay was performed. The IC_50_ value of human DYRK2 was greater than 10 μM (data not shown). In particular, KS-40070 showed strong specific inhibition against DYRK1B and relatively high selectivity against DYRK2.

### 3.3 Anti-adipogenic effects of KS-40070 in 3T3-L1 cells and ADMSCs

**FIGURE 3:**
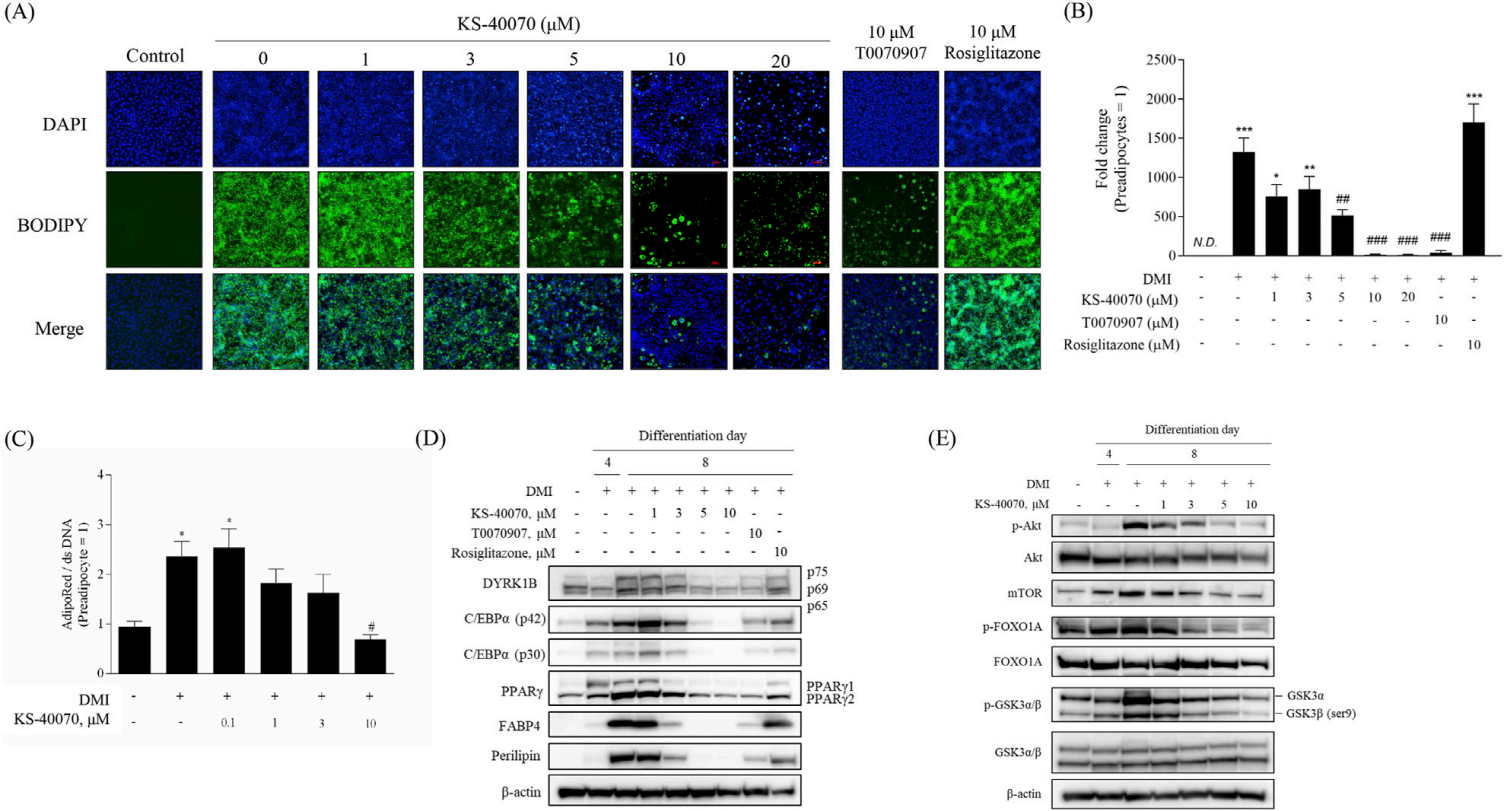
Anti-adipogenesis effect of a novel DYRK1B inhibitor, KS-40070. **(A)** Effect of KS-40070 on lipid accumulation in 3T3-L1 cells. Fluorescence images of lipid droplets (BODIPY, green) and nuclei (DAPI, blue) in 3T3-L1 cells (100✕ magnification). **(B)** Quantification of fluorescence intensities was performed using ImageJ software (green/blue). **(C)** Effect of KS-40070 on lipid accumulation in 3D ADMSCs. Results are presented as mean ± S.E.M. (n = 3) of at least three independent experiments. ^*^ *P* < 0.05 vs., ^***^ *P* < 0.001 vs. DMI (−) (preadipocytes); ^*#*^ *P* < 0.05, ^*##*^ *P* < 0.01, ^*###*^ *P* < 0.001 vs. DMI (+) (adipocytes). **(D,E)** Expression level of DYRK1B and other adipogenesis markers in 3T3-L1 cells as assayed by Western blot.

The dose-dependent effects of KS-40070 on lipid droplet accumulation in 3T3-L1 cells were investigated by staining lipid droplets with BODIPY and nuclei with DAPI. The differentiation of preadipocytes into adipocytes was confirmed, and lipid droplet accumulation was effectively inhibited at 10 μM of KS-40070 (Figures 3A,B). In addition, we analyzed the effects of KS-40070 on lipid accumulation in 3D human ADMSCs by staining lipids with AdipoRed and dsDNA with Hoechst 33,342 (Figure 3C).

Western blot was performed to determine whether KS-40070 affects the expression of DYRK1B and other adipogenesis-related markers. KS-40070 reduced the expression of DYRK1B and other adipogenesis transcription factors, such as PPARγ and C/EBPα, in a dose-dependent manner and adipocyte-specific markers were also effectively decreased at 5 μM (Figure 3D).

Our experimental data suggest that KS-40070 regulates FOXO1A via the inactivation of Akt (Figure 3E). Treatment with KS-40070 reduced the expression of phospho-Akt. Akt regulates adipogenesis through the inactivation and phosphorylation of several downstream targets. KS-40070 also reduced the expression of mTOR, phospho-GSK-3β, and phospho-FOXO1A, which are downstream substrates of Akt, in 3T3-L1 adipocytes. FOXO1A acts as an inhibitor in the adipogenic differentiation process. Akt-dependent FOXO1A phosphorylation promotes the translocation of FOXO1A from the nucleus to the cytoplasm, thereby suppressing its adipogenesis-inhibiting effect (Gross et al., 2008; Chen et al., 2018). KS-40070 inhibits the nuclear-cytoplasmic translocation of FOXO1A through the inactivation of Akt. Consequently, FOXO1A remains localized to the nucleus, where it suppresses adipogenic transcription factors such as PPARγ and C/EBPα.

### 3.4 Effect of KS-40070 treatment on lipid accumulation in DYRK1B knock-downed cells

**FIGURE 4:**
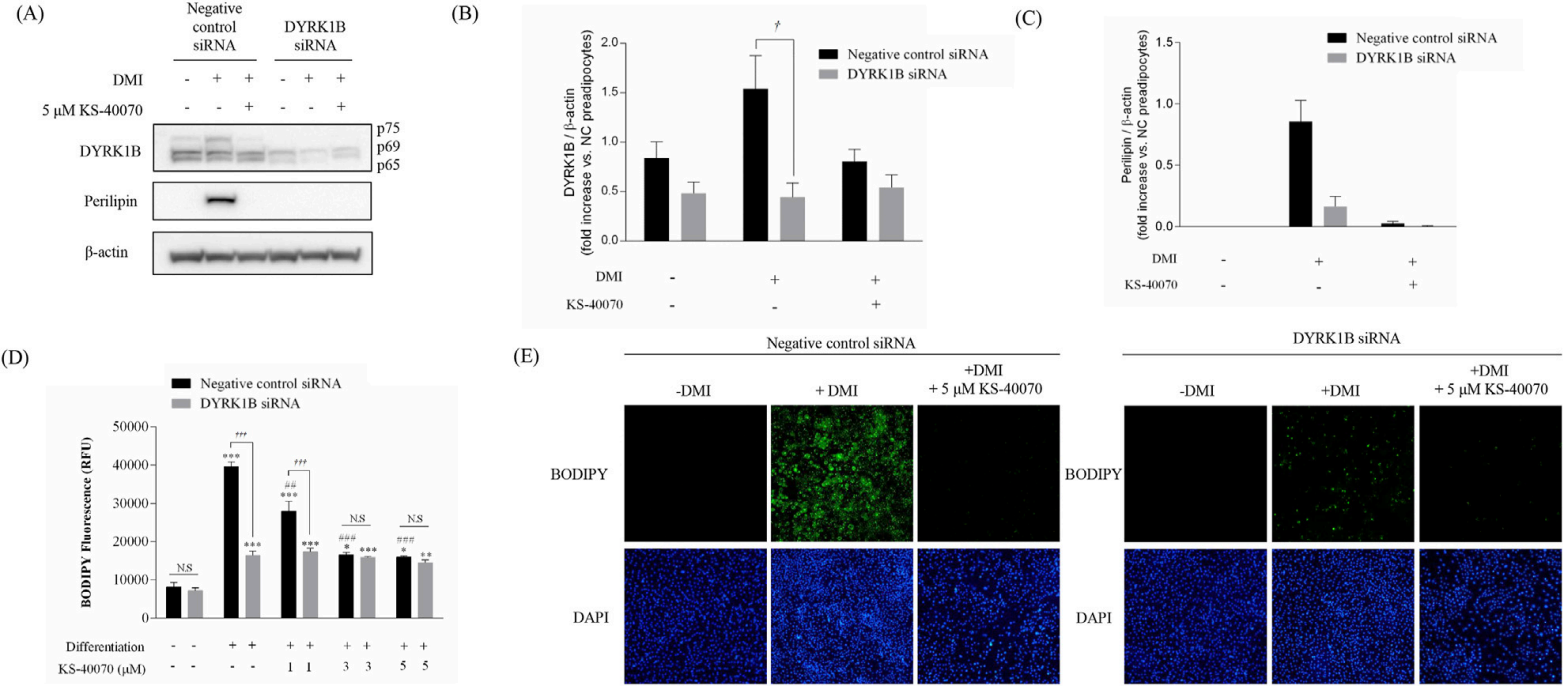
Knockdown effect by DYRK1B siRNA on lipid accumulation in 3T3-L1 cells. **(A)** Western blot analysis shows DYRK1B and lipid-droplet related proteins in cells with DYRK1B siRNA treatment and KS-40070. **(B)** DYRK1B protein levels relative to β-actin. **(C)** Perilipin protein levels relative to β-actin. Band intensities quantified using ImageJ. **(D)** Lipid staining with BODIPY, with fluorescence intensity measurement. **(E)** Fluorescence microscopy images of lipid droplets (BODIPY) and nuclei (DAPI) at ×100 magnification. Results are presented as mean ± S.E.M. (n = 3) of at least three independent experiments. ^*^ *P* < 0.05, ^**^ *P* < 0.01, ^***^ *P* < 0.001 vs. DMI (−) (preadipocytes); ^*##*^ *P* < 0.01, ^*###*^ *P* < 0.001 vs. DMI (+) (adipocytes); ^*†*^P < 0.05, ^*†††*^P < 0.001 vs. negative control siRNA transfected cells.

To verify whether the anti-obesity effect of KS-40070 is the result of specifically inhibiting DYRK1B, a siRNA-based knock-down assay was performed to specifically inhibit DYKR1B expression (Figure 4). DYRK1B siRNA selectively inhibited DYRK1B protein expression, and Perilipin expression level, as an adipogenic differentiation marker, was also extremely reduced in DYRK1B targeted siRNA-transfected cells (Figures 4A–C). The effect of DYRK1B inhibition on anti-adipogenesis was also demonstrated by lipid vesicle staining using BODIPY (Figures 4D,E). In the case of negative siRNA cells, lipid vesicles increased by induction of adipogenic differentiation, but in the case of DYRK1B specific siRNA-transfected cells, lipid vesicles were hardly produced even when adipogenic induction occurred. This anti-adipogenic effect was confirmed in the same way in cells treated with KS-40070, and these results show that the decrease in DYRK1B expression is related to anti-adipogenesis, and that the anti-adipogenic effect of KS-40070 is also a phenomenon that appears by targeting DYRK1B.

### 3.5 Effect of KS-40070 on body weight gain and plasma lipid profiles in DIO mice

Pharmacokinetic studies were performed to inform the design of *in vivo* DIO studies (data not shown). KS-40070 was absorbed at a T_max_ (time for C_max_) of 0.5 ± 0 h and C_max_ (maximum plasma concentration) of 0.069 ± 0.026 h. T_1/2_ (terminal half-life) was approximately 7 h. The AUC ∞ value (area under the plasma concentration time curve from time to zero) was 0.826 ± 0.082 μg h/mL in the intravenous administration group, and 0.0123 ± 0.05 μg h/mL in the oral administration group; the resulting F_t_ (oral bioavailability) was 12.4%. As such, DIO mice received twice-daily oral administrations of the experimental or control compounds in the DIO study.

**FIGURE 5:**
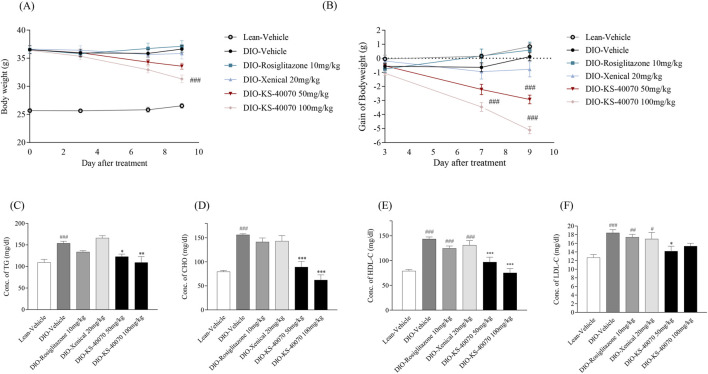
Effect of KS-40070 on body weight and plasma lipid profiles in DIO mice. Drugs were orally administered twice a daily at specific times. Body weight and body weight gain were measured twice a day for 9 days. Also, serum lipid profiles were measured using an automated biochemical analyzer **(A)** Changes in body weight **(B)** body weight gain **(C)** Changes of triglycerides concentration **(D)** Changes of total cholesterol concentration **(E)** Changes of HDL concentration **(F)** Changes of LDL concentration. Results are presented as mean ± S.E.M. (n = 7/group). ^*#*^ *P* < 0.05, ^*##*^ *P* < 0.01, ^*###*^ *P* < 0.001 vs. Lean group; ^*^ *P* < 0.05, ^**^ *P* < 0.01, ^***^ *P* < 0.001 vs. DIO-vehicle group.

In DIO mice study, the drugs were administered for 9 days twice daily and body weight and food intake were measured twice weekly. KS-40070 groups were 50 mg/kg and 100 mg/kg and 10 mg/kg rosiglitazone and 20 mg/kg Xenical were treated as positive controls. Body weights in the rosiglitazone and Xenical group showed no change. However, body weight gain of the KS-40070 treated groups were reduced in a dose-dependent manner (Figures 5A,B).

Cholesterol, triglycerides, HDL and LDL cholesterol level were measured to determine whether KS-40070 could improve plasma lipid profiles. In the vehicle group of DIO mice, all of these values were significantly increased. The rosiglitazone treated group showed slightly decreased plasma lipid profiles in DIO mice; the Xenical-treated group showed no changes. The KS-40070 treated groups, however, demonstrated significantly improved plasma lipid profiles such as cholesterol, triglycerides and LDL-cholesterol (Figures 5C–F).

### 3.6 Effect of KS-40070 on fat weight and adipocyte size in DIO mice

**FIGURE 6:**
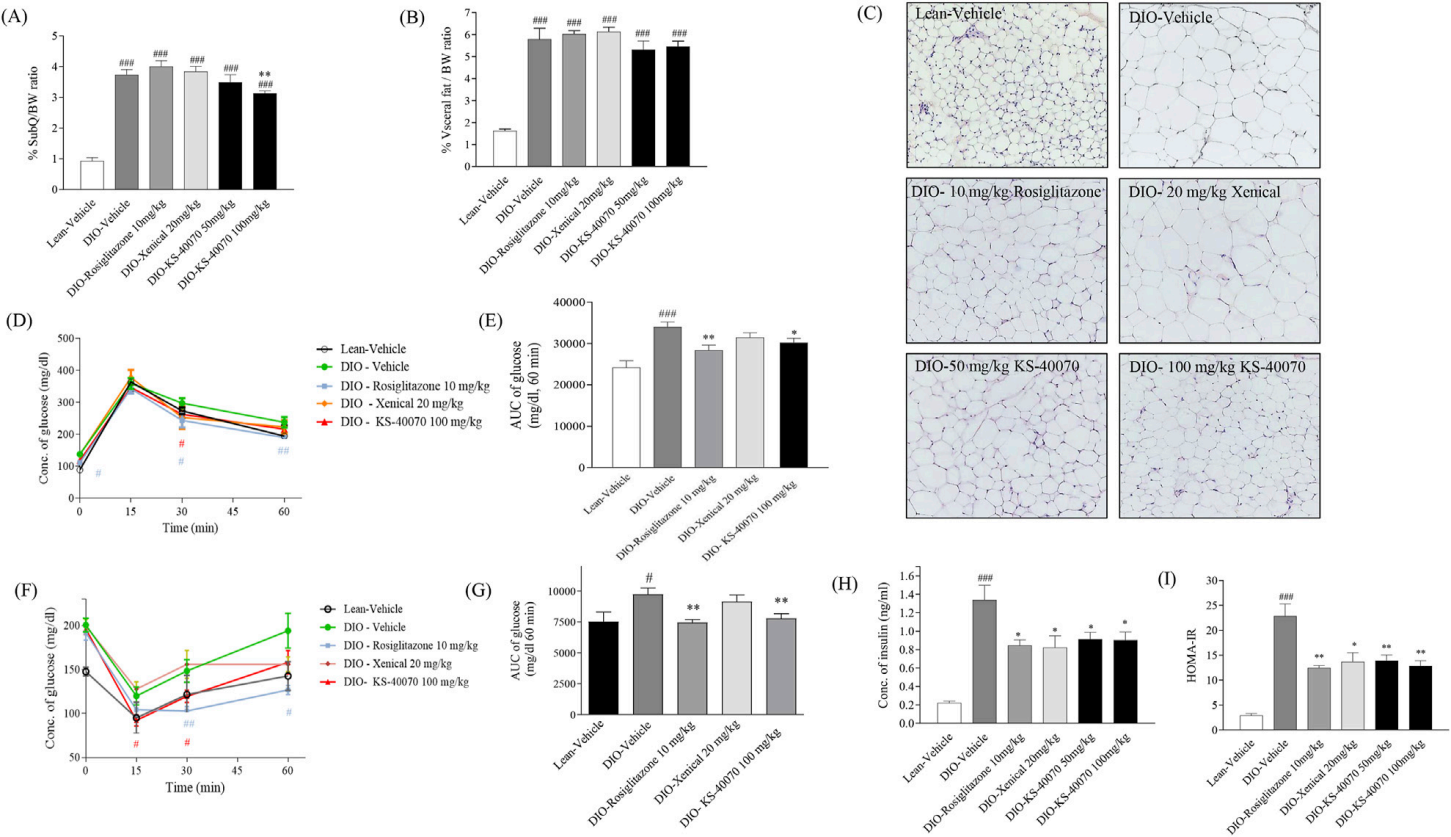
Effect of KS-40070 on fat weight, adipocyte size, OGTT, ITT and insulin resistance in DIO mice. **(A,B)** Visceral and subcutaneous fat weights were measured after mice were sacrificed. **(C)** Histological analysis of subcutaneous fat. Nuclei were stained with hematoxylin and cytoplasm with eosin. Images were obtained using a light microscope at 200✕ magnification. **(D)** Time-course changes in blood glucose levels during OGTT after oral administration of glucose and **(E)** Area under the curve (AUC) analysis for OGTT in each group **(F)** Time-course changes in blood glucose levels during ITT after intraperitoneal administration of insulin and **(G)** Area under the curve (AUC) analysis for ITT in each group **(H)** Plasma insulin concentrations and **(I)** HOMA-IR value in fasting status. Results are presented as mean ± S.E.M. (n = 7/group). ^#^ *P* < 0.05, ^*###*^ *P* < 0.001 vs. Lean group; ^*^ *P* < 0.05, ^**^ *P* < 0.01 vs. DIO-vehicle group.

To investigate whether KS-40070 decreased fat weight in DIO mice, subcutaneous and visceral fat weights were measured after mice were sacrificed. In 100 mg/kg KS-40070 treated group, subcutaneous fat weights were significantly decreased compared to vehicle group (Figures 6A,B). These results coupled with body weight change results suggest that reduction of fat weight is associated with body weight loss. At this time, subcutaneous fat weight was decreased more than visceral fat weight in KS-40070 treated group. Therefore, we performed a histological examination of the subcutaneous fat to evaluate the change in the size of adipocytes. Reference compound, Xenical-treated group had no effect, but the KS-40070 treated group reduced the size of subcutaneous fat cells (Figure 6C).

### 3.7 Effect of KS-40070 on oral glucose tolerance and insulin resistance in DIO mice

We assessed whether KS-40070 suppression of DYRK1B restore glucose tolerance and insulin resistance in DIO mice after oral treatment with 50 or 100 mg/kg, twice daily for 9 days. In the oral glucose tolerance test, plasma glucose levels as determined by the AUC of the glucose concentration curve were significantly reduced after administration of 10 mg/kg rosiglitazone and 100 mg/kg KS-40070 compared to vehicle (Figures 6D,E). Using the same model, we also measured KS-40070 effects on insulin sensitivity with an insulin tolerance test (Figures 6F,G). Plasma fasting insulin concentrations and HOMA-IR index of KS-40070 treated mice were significantly lower than those of the DIO-vehicle mice (Figures 6H,I).

### 3.8 Effect of KS-40070 on lipid accumulation and fatty liver-related markers expression in liver of DIO mice

**FIGURE 7:**
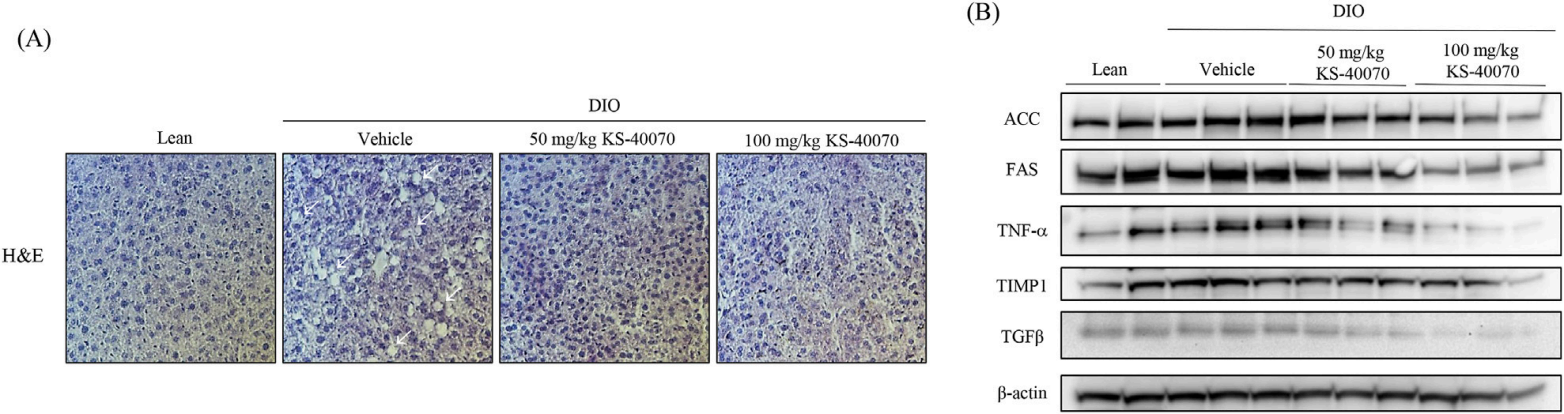
Effect of KS-40070 on lipid accumulation and fatty live-related markers expression in liver of DIO mice. **(A)** H&E analysis of livers. Images were obtained using a light microscope at 400✕ magnification. Red circles indicate hepatic lipid accumulation **(B)** Effect of KS-40070 on fatty liver related markers expression in liver of DIO mice.

To analyze the potential hepatotoxicity of KS-40070, the weight of the mouse liver and H&E staining were performed after autopsy. There were no differences in organ appearance and AST/ALT ratios among the groups (data not shown). H&E staining results showed that lipid accumulation was increased in the DIO-vehicle group (arrows), and hepatic lipid accumulation was reduced by KS-40070 administration (Figure 7A). Consistently, Western blot analysis was employed to investigate the impact of KS-40070 on the expression of fatty liver-related markers. The results demonstrated a dose-dependent decrease in the expression of fatty liver-related markers, including FAS, TNFα, TIMP1, and TGFβ, following treatment with KS-40070 (Figure 7B). These markers are known to be involved in various aspects of fatty liver pathogenesis, including lipid metabolism, inflammation, and fibrosis (Guo et al., 2022; Kotlyarov, 2023; Vachliotis and Polyzos, 2023). Thus, these results suggest that KS-40070 shows promise as a potential therapeutic agent for the treatment of non-alcoholic fatty liver disease (NAFLD) and related metabolic disorders without inducing hepatotoxic effects.

### 3.9 Homology modeling and molecular docking studies

**FIGURE 8:**
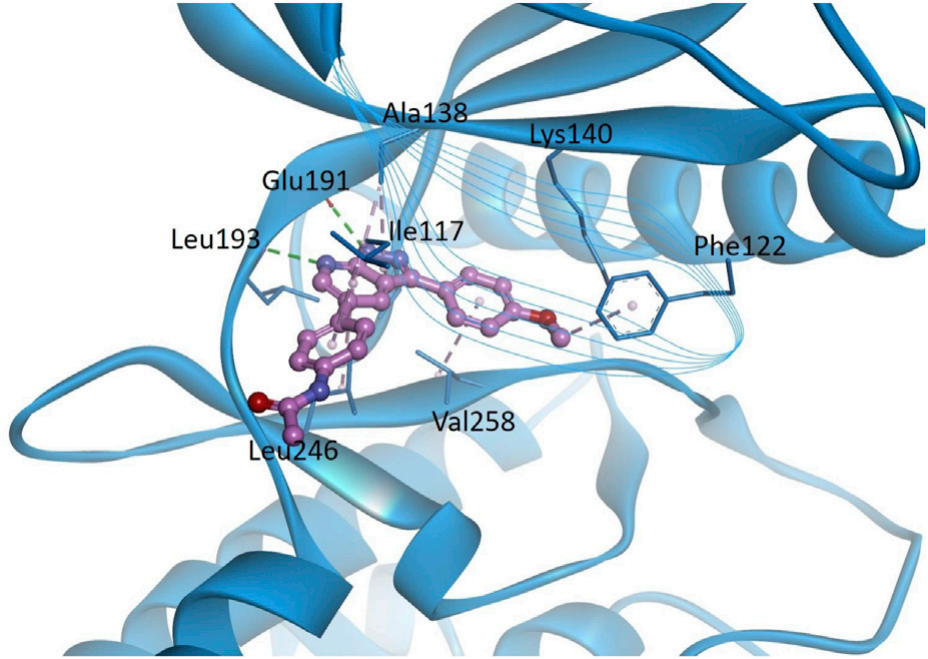
Docking models of KS-40070 for DYRK1B. Binding of KS-40070 (pink colored ball and stick) to the DYRK1B (blue colored ribbon). For clarity, key binding site residues are shown in sticks and labeled using the 3-letter amino acid code. The hydrogen bonds are displayed as green dashed lines and hydrophobic interactions are shown as pink dashed lines.

In order to predict the binding modes of KS-40070 for DYRK1B, we performed molecular docking studies with DYRK1B homology model (Figure 8). The pyrazolopyrimidine ring binds to the hinge region, which forms two hydrogen bonds with Glu191 backbone CO and Leu193 backbone NH, and hydrophobic interactions with Ala138, Val174 and Leu246. Phenylacetamide group is oriented towards solvent exposed area by forming hydrophobic interaction with Ile117. 4-methoxyphenyl ring forms hydrophobic interactions with Val258 and Phe122.

## 4 Discussion

The results of our study shed light on the potential therapeutic effects of KS-40070, a novel selective DYRK1B inhibitor, in the context of metabolic disorders such as obesity, diabetes, and fatty liver disease.

DYRK1B, a serine/threonine kinase also known as minibrain-related kinase (Mirk), belongs to the DYRK family and has been implicated in various diseases. Notably, DYRK1B is found to be overexpressed in certain cancers, and inhibitors targeting DYRK1B have shown promising therapeutic effects in cancer treatment (Chen et al., 2017; Becker, 2018). Additionally, DYRK1B has emerged as a significant player in metabolic syndrome (Keramati et al., 2014; Mirshahi et al., 2014). Studies have revealed associations between DYRK1B mutations and metabolic disorders such as coronary artery disease, hypertension, and diabetes (Keramati et al., 2014). In particular, recently studies suggest that DYRK1B expression is noticeably increased during adipogenesis in 3T3-L1 cells and is also involved in lipogenesis, regulation of gluconeogenic enzymes, glucose-6-phosphatase, weight gain, appetite, and metabolic syndrome (Keramati et al., 2014). Additionally, inhibition of DYRK1B has been shown to reduce adipogenesis, indicating its potential as a therapeutic target for the treatment of obesity in individuals with metabolic syndrome. These findings highlight the multifaceted role of DYRK1B in disease pathogenesis and highlight its therapeutic potential in both cancer and metabolic disorders (Masaki et al., 2015). Like these findings, our research demonstrates the importance of DYRK1B in adipogenesis. Through Western blot analysis, we observed a gradual increase in DYRK1B expression during adipogenesis in 3T3-L1 cells, showing that 3T3-L1 preadipocytes contain p65 and p69 of DYRK1B, and that p75 is induced in late adipogenesis (Leder et al., 2003). Additionally, subsequent gene silencing analysis revealed that knockdown of DYRK1B resulted in decreased expression of Perilipin, a key protein involved in lipid droplet formation (Itabe et al., 2017), suggesting a role for DYRK1B in lipid formation and its potential impact on obesity. Recently published paper identified DYRK1B as a biomarker of hepatic gluconeogenesis through omics-based analysis (Li et al., 2025). However, further investigation using expanded omics approaches will be necessary to elucidate the broader roles and functions of DYRK1B in various cellular-stress pathways relevant to metabolic disorders, as well as its potential as a diagnostic or prognostic biomarker (Tang et al., 2023; Liang et al., 2024).

Additionally, our study presents KS-40070 as a potent inhibitor of DYRK1B. In our previous study, we synthesized a series of 1H-pyrazolo [3,4-b]pyridine derivatives as DYRK1B inhibitors (Park et al., 2021), and KS-40070 of these derivatives showed strong inhibitory potency (IC_50_ value of 18 nM) against DYRK1B; It exhibited relatively high selectivity toward other members of the DYRK family. KS-40070 dose-dependently inhibits lipid droplet accumulation in 3T3-L1 cells and human ADMSCs, indicating its potential as a therapeutic agent to inhibit differentiation into obesity-related adipocytes.

Moreover, our study provides molecular pathway into the action of KS-40070 in regulating adipogenesis. Our experimental data suggest that KS-40070 modulates the activity of FOXO1A through the inhibition of Akt, a pivotal signaling pathway implicated in adipogenesis regulation. The observed reduction in phospho-Akt levels following KS-40070 treatment is consistent with its proposed mechanism of action. Akt signaling is known to promote adipocyte differentiation and lipid accumulation by activating downstream targets such as mTOR, GSK-3β, and FOXO1A (Park et al., 2014; Savova et al., 2023). Notably, our results indicate a decrease in the expression of phospho-mTOR, phospho-GSK-3β, and phospho-FOXO1A upon KS-40070 treatment, suggesting attenuation of Akt signaling cascade. Of particular significance is the role of FOXO1A in mediating the effects of KS-40070 on adipogenesis. FOXO1A is a transcription factor involved in various cellular processes, including adipocyte differentiation (Nakae et al., 2003) Our findings demonstrate that KS-40070 inhibits the nuclear-cytoplasmic translocation of FOXO1A, thereby modulating its activity. Consequently, the expression of adipogenic transcription factors such as PPARγ and C/EBPα, which are downstream targets of FOXO1A, is suppressed by KS-40070 treatment. Additionally, our study utilized DYRK1B knockdown experiments to confirm that the anti-adipogenic effects of KS-40070 are mediated through DYRK1B inhibition. Transfection of DYRK1B RNAi oligonucleotides resulted in reduced DYRK1B protein expression, and subsequent treatment with KS-40070 showed no effect on lipid accumulation in DYRK1B-knockdown cells. This observation provides compelling evidence that the anti-adipogenic effects of KS-40070 are mediated through DYRK1B inhibition. These findings have important implications for translational medicine, particularly in the context of developing targeted therapies for metabolic syndrome. The selective inhibition of DYRK1B by KS-40070, coupled with its demonstrated efficacy in both 2D and 3D adipogenic models, represents a promising therapeutic strategy grounded in mechanistic insight. Recent advances in omics technologies and high-content screening have increasingly enabled the identification of disease-specific molecular targets such as DYRK1B (Huang et al., 2024; Li et al., 2025), and our study contributes to this trend by functionally validating DYRK1B as a regulator of adipogenesis and glucose homeostasis. Furthermore, the use of human ADMSCs in 3D culture systems enhances the clinical relevance of our findings, bridging the gap between basic molecular discovery and future therapeutic application.

*In vivo* DIO study, we examined the potential cytotoxicity of KS-40070 in various mammalian cell lines and our data is suggesting that KS-40070 did not induce any cytotoxicity among the cells we have tested. Next, we evaluated the pharmacokinetic characteristics of KS-40070 in oral versus intravenous administration. Following intravenous administration at 5 mg/kg, KS-40070 showed high systemic CL (6.09 ± 0.573 L/h/kg) and high V_ss_ (9 ± 2 L/kg); also, more than 50% of the compound was detectable after 7 h. Upon oral administration, KS-40070 was absorbed quickly (T_max_ = 0.5 h) and, as with intravenous administration, more than 50% of KS-40070 was detectable after 7 h. The oral bioavailability of KS-40070 was found to be 12.4%. Based on these results, DIO mice were orally administered KS-40070 twice daily at specific times for 9 days.

DIO mice are characterized by obesity, glucose intolerance, insulin resistance, and mild hyperglycemia (Lang et al., 2019). In the present study, KS-40070 was administered to DIO for 9 days. The reference compounds were rosiglitazone, a PPAR γ agonist, and Xenical, anti-obesity drug (Vickers et al., 2011; Ribas-Latre et al., 2019). Beginning from the 7th day of drug administration, mice treated with KS-40070 exhibited significant reductions in body weight accompanied by a decrease in food intake, indicating a potential anti-obesity effect. Furthermore, KS-40070 treatment led to improvements in plasma lipid profiles, including reductions in triglycerides, cholesterol, LDL, and HDL, along with a decrease in subcutaneous and visceral fat deposition. In this study, as appetite was reduced through changes in feed intake, it is expected that fatty acids were transported to the liver and muscles, causing a beta-oxidation process (Chu L. et al., 2022). DYRK1B inhibitors can suppress fat synthesis in adipocytes and promote fat breakdown. This may contribute to the reduction of body fat and weight loss (Kokkorakis and Gaitanou, 2020). Also, DYRK1B inhibitors can enhance insulin signaling in liver and muscle cells, thereby improving insulin sensitivity. This can have a positive effect on blood glucose regulation (Bhat et al., 2022). Additionally, histological examination revealed a decrease in lipid droplet sizes in adipose tissue, indicative of reduced adiposity in KS-40070-treated mice. Moreover, KS-40070 administration demonstrated beneficial effects on glucose tolerance and insulin resistance. Specifically, KS-40070-treated mice exhibited reductions in glucose levels during OGTT, as well as decreased fasting insulin levels. Furthermore, the homeostatic model assessment of insulin resistance index was significantly lowered in KS-40070-treated mice, indicating improved insulin sensitivity. Histological analysis demonstrated that hepatic lipid accumulation was reduced by KS-40070. Additionally, the expression of lipid accumulation and inflammatory markers (ACC, FAS, TNFα, TIMP1 and TGFβ) in the liver was also decreased. These results indicate that KS-40070 reduces hepatic lipid accumulation and suppresses inflammatory markers, suggesting its potential to alleviate fatty liver disease. The downregulation of ACC, FAS, TNFα, TIMP1, and TGFβ indicates improved lipid metabolism, reduced inflammation, and fibrosis suppression. These effects may help prevent the progression of NAFLD and maintain overall liver function.

Also, we performed molecular docking of KS-40070 with the DYRK1B homology model to predict their binding modes. The docking results suggest that KS-40070 interacts with the hinge region via hydrogen bonding with Glu191 and Leu193, along with additional hydrophobic interactions with Ala138, Val174, and Leu246. Previous studies have shown that DYRK1B inhibition can ameliorate endothelial dysfunction (Pramotton et al., 2023) and chronic inflammatory diseases (Ciurus et al., 2025). In contrast, DYRK1B also plays a critical role in neuronal system development, particularly in the central nervous system, by regulating astrocyte differentiation and maintaining cellular homeostasis (He et al., 2018). Given these diverse physiological functions, the development of DYRK1B-targeted therapeutics must carefully consider mutation-specific effects and disease contexts to maximize therapeutic efficacy while minimizing potential off-target effects.

## 5 Conclusion

Our study demonstrates that KS-40070, a selective DYRK1B inhibitor, has significant therapeutic potential for treating metabolic disorders such as obesity, type 2 diabetes, and fatty liver disease. KS-40070 effectively suppresses adipogenesis by inhibiting key adipogenic proteins and pathways, without inducing cytotoxicity. *In vivo* studies in DIO mice showed reductions in body weight, fat mass, and improved insulin sensitivity and lipid profiles. Additionally, KS-40070 mitigated liver inflammation and fibrosis. These findings indicate that the selective DYRK1B inhibitor could be a promising new treatment for metabolic syndrome as obesity, type 2 diabetes and fatty liver. Nonetheless, to fully evaluate the potential of KS-40070 for development as a treatment for obesity and type 2 diabetes, it is necessary to consider not only species-specific differences in efficacy and drug metabolism, but also physiological issues arising from inter-species differences. *In vitro* based experiments and *in vivo* mouse models provide important preliminary insights, but to bridge the gap to clinical application, larger animal models and human-derived systems capable of evaluating efficacy and pharmacokinetics, as well as validation in clinical trials, will be needed.

## Data Availability

The data generated for this study are available on request to the corresponding author.
